# Gossip-Based Solutions for Discrete Rendezvous in Populations of Communicating Agents

**DOI:** 10.1371/journal.pone.0112612

**Published:** 2014-11-14

**Authors:** Christopher D. Hollander, Annie S. Wu

**Affiliations:** Department of Electrical Engineering and Computer Science, University of Central Florida, Orlando, FL, United States of America; University of Westminster, United Kingdom

## Abstract

The objective of the rendezvous problem is to construct a method that enables a population of agents to agree on a spatial (and possibly temporal) meeting location. We introduce the *buffered gossip algorithm* as a general solution to the rendezvous problem in a discrete domain with direct communication between decentralized agents. We compare the performance of the buffered gossip algorithm against the well known *uniform gossip algorithm*. We believe that a buffered solution is preferable to an unbuffered solution, such as the uniform gossip algorithm, because the use of a buffer allows an agent to use multiple information sources when determining its desired rendezvous point, and that access to multiple information sources may improve agent decision making by reinforcing or contradicting an initial choice. To show that the buffered gossip algorithm is an actual solution for the rendezvous problem, we construct a theoretical proof of convergence and derive the conditions under which the buffered gossip algorithm is guaranteed to produce a consensus on rendezvous location. We use these results to verify that the uniform gossip algorithm also solves the rendezvous problem. We then use a multi-agent simulation to conduct a series of simulation experiments to compare the performance between the buffered and uniform gossip algorithms. Our results suggest that the buffered gossip algorithm can solve the rendezvous problem faster than the uniform gossip algorithm; however, the relative performance between these two solutions depends on the specific constraints of the problem and the parameters of the buffered gossip algorithm.

## Introduction

We introduce a solution to the rendezvous problem when there are a finite number of discrete meeting locations and the rendezvous process occurs in a decentralized multi-agent system where agents are able to directly communicate with their local neighbors. Decentralized environments offer a degree of simplicity, scalability, and robustness to error that cannot be easily obtained with a centralized approach [1], [2]. We assume the special case that all meeting locations are equally preferable to one another.

The *decentralized* rendezvous problem is a specific instance of the consensus problem [3]–[8]. In the decentralized rendezvous problem, one assumes that there is a population of leaderless agents that want to rendezvous, but each agent initially wants to rendezvous at a different location. The objective of the decentralized rendezvous problem is to construct a method that enables a population of agents to form a consensus on a spatial (and possibly temporal) meeting location without the help of a centralized control mechanism.

Solutions to the decentralized rendezvous problem can be broken down into four categories based on location type (continuous coordinates or discrete locations) and communication scheme (*direct* or *indirect*). Direct communication occurs when two agents can directly communicate with one another; e.g. using a point-to-point protocol. Indirect communication occurs when one agent broadcasts information and another agent happens to be in range and receives it, when one agent modifies the environment in some way and another agent interprets the modification as information (i.e. stigmergy), or when one agent observes its neighboring agents in order to acquire information.

Previous authors have studied three of these four solution categories: rendezvous in the continuous domain with indirect communication between agents [4], [5], [7]; rendezvous in the discrete domain with indirect communication between agents [3], [7]; and rendezvous in the continuous domain with direct communication between agents [6], [8]. In this paper, we study the fourth category of solution: rendezvous in the discrete domain with direct communication between agents. Rendezvous in this fourth category is most likely to occur in autonomous multi-agent systems; for example, autonomous vehicles that need to gather at a specific waypoint instead of an arbitrary location. To the best of our knowledge, this category has not been studied specifically, but the general idea has been examined in the context of information dissemination [1], [9], [10], consensus formation [11], [12], and opinion dynamics [13].

Our primary contribution is the introduction of the *buffered gossip algorithm* as a solution in the fourth category: rendezvous in the discrete domain with direct communication between agents. Agents using the buffered gossip algorithm transmit rendezvous information to one randomly selected neighbor at a time, and store incoming rendezvous information in a buffer that is periodically reset. This buffer allows agents to use multiple information sources when determining their desired rendezvous location. An internal clock controls the rate at which an agent updates its desired rendezvous location and resets its buffer. Because of the buffer, the buffered gossip algorithm is particularly suited to scenarios where it is not possible (or not desired) for an agent to access, process, and re-transmit existing data prior to the introduction of new data, or when agents are capable of receiving and decoding multiple transmissions simultaneously. Such a scenario may be imagined in certain types of multi-agent surveillance systems, or when it is not practical to synchronize the actions of a population. From a practical stand point, it is reasonable to assume that many systems are unable to respond at a speed required for agents to update their state in response to every received transmission prior to the reception of new information.

To prove that the buffered gossip algorithm is a solution to the decentralized rendezvous problem, we derive the conditions under which a consensus can be formed on rendezvous location. We also show that this consensus forms in the presence of noise and agent failure, and once formed remains stable until the system is disturbed by external forces.

We also contribute an empirical comparison between the buffered gossip algorithm and the well known *uniform gossip algorithm* [9], [10]. Agents using the uniform gossip algorithm transmit rendezvous information to a neighbor that has been selected according to a uniform distribution, and that neighbor then immediately updates its own desired rendezvous location to match the newly received information. Because the uniform gossip algorithm does not use a buffer, it is only capable of storing a single piece of information at any given time. It is our expectation that, in most situations, the use of a buffer will allow the buffered gossip algorithm to solve the decentralized rendezvous problem faster than the uniform gossip algorithm.

We begin our introduction to the buffered gossip algorithm by discussing previous research that is related to the topic of rendezvous in the discrete domain with direct communication. Following this discussion, we describe the notational conventions used in our equations and define the buffered gossip algorithm. We then derive the conditions under which the buffered gossip algorithm solves the decentralized rendezvous problem. We present the uniform gossip algorithm in the same framework as the buffered gossip algorithm and show it too can be used to solve the decentralized rendezvous problem under the same conditions as a buffered approach. Finally, we use a multi-agent simulation to conduct a series of experiments that compare the rendezvous time between the buffered gossip algorithm and uniform gossip algorithm.

## Related Work on the Consensus Problem

The decentralized rendezvous problem that we study in this article is a specific instance of the *decentralized consensus problem*. The objective of the decentralized consensus problem is to design a method that enables agents to communicate and exchange information such that, in finite time, every agent adopts the same value without using a centralized control mechanism [6], [14], [15]. Our research imposes the additional constraint that the values of interest are discrete, and cannot be averaged together or otherwise recombined.

Gossip algorithms [16]–[21] and leader election algorithms [12], [22], [23] are two popular approaches to solving the decentralized consensus problem, and either algorithm has the potential to solve the decentralized rendezvous problem in the discrete domain. Both of these algorithms define how agents receive, process, and transmit information in a decentralized environment, but they use different philosophies to induce a consensus.

Solutions to the decentralized consensus problem that use gossip algorithms depend on randomness to slowly drive a system towards consensus. Agents using a gossip algorithm contain a *state value*, a *gossip mechanism*, and a *gossip protocol*. The state value stores the information being spread through the network. The gossip mechanism determines how the agent selects the target(s) for its transmission. Traditionally, selection of transmission targets is done uniformly and at random, but there is no strict requirement for this practice. Three general gossip mechanisms are used in the existing literature: select a single target from the local neighborhood [10], [15], [24], [25], select a single target from the entire network [1], [2], [16]–[18], [26]–[29], or select multiple targets from the local neighborhood [10], [30]. We are primarily interested in the case where a single target is selected from the local neighborhood due to our focus on the direct communication. The gossip protocol determines what the contents of a transmission will be, and how the receiver of a transmission will use the new information to update their internal state. The specific implementation of the gossip protocol depends on the problem being solved. With respect to the decentralized rendezvous problem in the discrete domain with direct communication, we are most interested in gossip protocols used for information dissemination [1], [9]–[11], [16], [21], [27], [31] (other common protocols include those for aggregation [19], [20], [32] and the construction of overlay networks [21], [25]). In protocols for information dissemination, the task is to design an algorithm that results in every agent having the same state value as quickly as possible. As information spreads, it can either replace the current information contained within an agent [10], [16], [21], or it can be stored alongside the existing information with the goal of having every agent aware of all other state values in the system [11], [21]. Common applications of information spread protocols include database synchronization [9], [16], balancing processor loads [1], [31], and accumulating information for use by other algorithms [11].

Solutions to the decentralized consensus problem that use leader election algorithms [12], [22], [23] depend on a single entity, called the leader, to dictate a consensus value to the rest of the population. The most well known and widely used leader election algorithm is Paxos [12], [22], although it is recognized that a real-world implementation of Paxos does not typically resemble the theoretical simplicity [23]. Agents that implement Paxos behave according to a predefined role. They can be either a *proposer*, an *acceptor*, a *leader*, or a combination of the three. A proposer transmits potential values for consensus to the acceptors. An acceptor chooses whether or not to accept the proposed value and lets the sender of that value know if it is accepted. If a majority of acceptors accept a proposed value, then the proposer of that value may become the leader. Learners determine the consensus value by receiving information from the acceptors and identifying the value accepted by a majority of acceptors. For a full description of how Paxos works, we refer the reader to the work of Lamport [12]. One of the biggest strengths of Paxos, besides its ability to form a consensus, is that it is fault tolerant. Leaders are selected based on a majority vote, so the failure of an agent to transmit does not stop the consensus process. Leaders can also be replaced in the event that they fail. Paxos has been primarily applied to database replication in IT systems [33], but more recently it has also been proposed for consensus formation in multiagent systems [34].

Despite their success in the literature on the consensus problem, both gossip algorithms and leader election algorithms have flaws that appear in a discrete domain when agents are allowed to communicate directly with one another. In the case of gossip algorithms, the issue of competition between values is largely neglected. The research on information dissemination when agents can only store a single value is primarily interested in the propagation speed, and it is often assumed that the systems start with only one agent containing that information; the rest are empty. In the decentralized rendezvous problem, as we study it, every agent is initialized with a different value, and those values must compete for dominance. It is unknown if the existing performance models for gossip algorithms continue to hold true in the presence of competing information. In the research that allows agents to build up information in every node, there is no certain way to know if and when every agent in a truly decentralized system has all of the information. So, there can be no guarantee that all agents will select the same value from among the information they are aware of. In large networks, this also requires that every agent maintain a large memory. In the case of leader election algorithms, the selection of a leader must occur before consensus is possible; this raises the question, “would it be faster just to use a different consensus algorithm to choose the rendezvous location, instead of first picking a leader and then having that leader propagate the value through the network by using an information dissemination algorithm?” Furthermore, many leader election algorithms rely on the ability of agents to broadcast information; in the specific problem that we study, agents do not possess this capability. Paxos, specifically, also requires that agents be able to respond to a transmission. This is a limitation that we do not assume in our study of the decentralized rendezvous problem.

Our solution, the *buffered gossip algorithm*, provides an abstraction layer for gossip algorithms that allows us to address these problems associated with competition, limited memory, an inability to broadcast, and a lack of transmission acknowledgement. The buffered gossip algorithm takes the structure of a gossip algorithm and incorporates the use of a buffer to temporarily store the state values from multiple neighbors. This buffer allows state values to compete with one another, while at the same time keeping the storage requirements of an agent proportional to the size of its local neighborhood. This buffer also allows agents to receive, store, and consider multiple transmissions when calculating a new state value, instead of simply updating to the latest information received from another agent. Additionally, because we build upon the gossip algorithm, we do not need to depend on broadcast communication or transmission acknowledgment, and so our solution inherits the same simplicity, scalability, and robustness that made gossip algorithms attractive to earlier researchers.

## Notation

For analytical purposes, we model a population of agents as a network; nodes represent agents and edges represent the communication/interaction links between those agents. Throughout this paper we indicate matrices and vectors with bold upper and lowercase symbols: 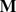 for matrices and 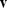 vectors. Individual elements will be indexed, non-bold, lowercase symbols: 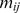 for matrices and 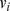 for vectors. The number of elements in an arbitrary set, 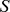, is denoted 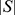. The probability of an arbitrary event, 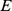, is denoted 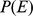.

## The Buffered Gossip Algorithm

The *buffered gossip algorithm* is derived from a *randomized gossip process*. To describe this derivation, we first define a randomized gossip process and then use this definition to introduce the buffered gossip algorithm.

### Randomized Gossip Processes

We define a randomized gossip process as an abstraction layer for gossip algorithms.

Gossip algorithms define how agents receive, process, and transmit information in a decentralized environment. In a traditional gossip algorithm, each node contains a *state value*, a *gossip mechanism*, and a *gossip protocol*. The gossip mechanism determines how a receiver is selected. The gossip protocol determines what the contents of a transmission will be, and how the receiver of a transmission will use the new information to update their internal state. In a traditional gossip algorithm, the internal clock of a node is driven by a *timing model*. This timing model is homogeneous across all nodes in the network (e.g. every node ticks according to an independent Poisson distribution with 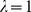).

Randomized gossip processes abstract gossip algorithms by treating each node as a self contained unit with an independent timing model, state value, gossip mechanism, and gossip process. A randomized gossip process also assumes that each node has a *buffer* and a *state update protocol*. As a result, it is possible for different nodes within the same network to use different timing models. The inclusion of a buffer means that a node can store multiple pieces of information and thus have an increased awareness of its environment. The state update protocol describes how to process the information in the buffer.

Let 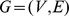 be an arbitrary network defined by a set of nodes, 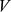, and a set of edges, 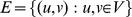, such that node 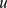 points to node 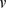. Let the neighbors of node 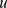 be defined as 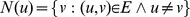. A randomized gossip process specifies how information is propagated over 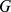 when each node, 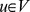, possesses a timing model, a state value, a buffer, a gossip mechanism, a gossip protocol, and a state update protocol. Using this definition, a gossip algorithm becomes a randomized gossip process that uses a specific timing model, gossip mechanism, gossip protocol, and state update protocol.

#### Timing Models

The timing model of node 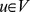 controls the rate at which node 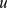 exchanges data with neighboring nodes in accordance with its gossip mechanism and the rate at which node 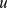 updates the state value 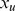 in accordance with its state update protocol. A timing model can either be *asynchronous* or *synchronous*. Under an asynchronous timing model, nodes activate independently of one another. For the purposes of analysis, we assume that every node in the network possesses a clock that ticks according to a Poisson process with rate 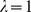. This is equivalent to a single clock that ticks according to a Poisson process with a rate of 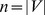 [20]. We call the instant of time during which these 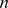 nodes act a *time step* and reserve the term *tick* to denote the advancement of a node's internal clock. One time step can be thought of as a discrete unit of time. In practice, this means that under an asynchronous timing model an average of 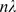 nodes are chosen independently and uniformly, at random, to transmit their information during each time step [20]; i.e. on average, one time step consists of 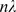 ticks. In the case of a synchronous timing model, the internal clock of each node is dependent on the clock of some other node in the network. Nodes using a synchronous timing model can be configured to activate sequentially, partially in parallel, or fully in parallel with each time step.

#### State Values

The state value of node 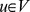 is defined as 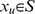 where 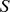 is the set of all possible state values. In the context of the decentralized rendezvous problem, these values are rendezvous locations.

#### Buffers

The buffer of node 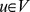 stores the data that node 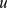 has received from 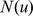 since the last tick of node 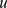. The buffer of node 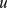 is defined as 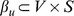 such that 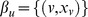 is a set of tuples of node 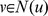 and the state value 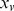 as seen by node 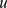. For example, 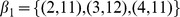 indicates that node 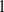 received the state value 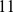 from nodes 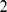 and 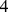 and the state value 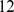 from node 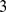. For convenience, we use 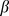 without a subscript to denote the set of all buffers in the network.

#### Gossip Mechanisms

The gossip mechanism of node 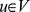 is a decision rule that determines which node(s) will receive 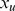 when node 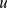 transmits. The gossip mechanism of a randomized gossip process selects one node from 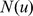 as the target for transmission. This node, 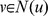, is selected at random. If 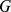 is not a weighted graph, then this selection process occurs according to a uniform distribution. If the edges of 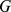 are weighted to reflect connection strength, then those weights can be used to derive an alternative distribution for the selection process. The implementation of such an alternative selection process is determined at the algorithmic level, with different implementations yielding different gossip algorithms.

#### Gossip Protocols

The gossip protocol of node 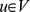 determines what will be transmitted to the selected neighbor, 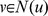, and what that neighbor will do with the new information once it has been received. The gossip protocol of a randomized gossip process transmits the value of 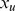 without modification and stores incoming information as a tagged pair within the buffer; if node 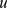 transmits 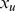 to a neighboring node, 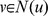, then 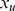 is stored in 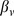 as the tuple 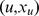.

#### State Update Protocols

The state update protocol of node 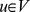, defined as 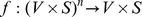, describes how 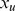 is derived from 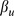. This derivation uses the companion functions 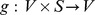 and 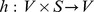 that extract the components of the tuple returned by 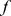. For example, if 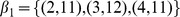 then one possible result is that 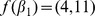, 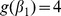, and 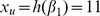.

Because a randomized gossip process is abstract, it does not specify the implementation details of a state update protocol. Instead, these details are specified at an algorithmic level, such that each definition of 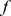 yields a unique gossip algorithm. For instance, the uniform gossip algorithm [10] is a randomized gossip process in which 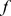 is defined to return the last value added to 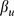.

State update protocols can be either *selection-based* or *aggregation-based*. In a section-based state update protocol, 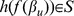. In an aggregation-based state update protocol, either 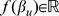 or 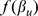 is an object constructed from multiple elements within 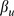.

### Buffered Gossip Algorithms

We define a *buffered gossip algorithm* as a randomized gossip process in which there is a positive probability that the buffer contains more than one piece of information (i.e. 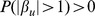). This may occur when a node receives multiple simultaneous transmissions from its neighboring nodes, or when a node accumulates information over a finite period of time.

The timing model of a node that uses a buffered gossip algorithm can be either asynchronous or synchronous. Our current research uses asynchronous timing models due to our focus on decentralized systems, and because it is often impractical to maintain the synchronization of large decentralized populations.

The gossip mechanism and gossip protocol used by a buffered gossip algorithm are identical to the gossip mechanism and gossip protocol used by a randomized gossip process. Each node transmits to only one neighbor at a time, and that neighbor is selected uniformly at random. Upon receiving a transmission, a node stores the associated information in its buffer along with the identification of sender.

Although there are many possible implementations of a state update protocol, our interest in the decentralized rendezvous problem drives us to focus on two specific selection-based state update protocols that ensure 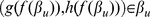: *proportional selection* (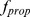) and *maximum frequency selection* (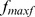). These selection-based state update protocols are based on two well known methods of information dissemination in opinion dynamics: the “voter model” [13] and the “label propagation algorithm” [35]. The implementation of each of these methods produces two distinct buffered gossip algorithms. Nodes that use a buffered gossip algorithm that implements the proportional selection protocol select a single element of 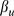, chosen uniformly at random and returns the associated state value. For example, if 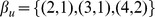 then 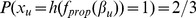 and 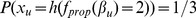. A buffered gossip algorithm using proportional selection is equivalent to a voter model [13], [32], [36]–[38] on a network with a time-varying topology. At any given time step, 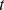, the neighborhood of each node, 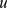, consists only of those nodes transmitting to node 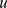. Nodes that use a buffered gossip algorithm that implements the maximum frequency selection protocol select a single element of 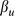, chosen such that 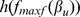 is the most frequently occurring state value in node 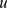
's buffer (with ties broken randomly) and 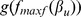 is a randomly chosen node associated with 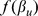. For example, if 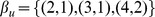 then 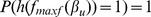 with 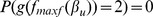 and 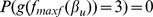 with the final result that 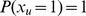. A buffered gossip algorithm using maximum frequency selection is equivalent to the Label Propagation Algorithm [32], [35] on a network with a time-varying topology. At any given time step, 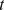, the neighborhood of each node, 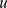, consists only of those nodes transmitting to node 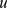.

Any buffered gossip algorithm that implements a specific timing model, gossip mechanism, gossip protocol, and state update protocol may capable of solving the decentralized rendezvous problem in the discrete domain with direct communication.

## Buffered Gossip Algorithms as a Solution to the Rendezvous Problem

To show that a buffered gossip algorithm can solve the decentralized rendezvous problem in the discrete domain with direct communication between agents, we first describe an analytical framework that allows us to study networks of gossiping nodes. We then use this framework to show that a buffered gossip algorithm will successfully solve the decentralized rendezvous problem when a network contains a directed spanning tree, and the nodes of that network employ both an asynchronous timing model and a selection-based state update protocol. Formally, we say that the network, 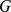, *contains* a directed spanning tree, 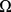, if 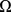 is a subgraph of 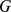. Next, we will discuss the impact of noise and node failure on the ability of a buffered gossip algorithm to form a consensus. Finally, we will show that once consensus is achieved, it remains in place until externally influenced. We do not investigate alternative timing models (e.g. synchronous) or non-selection based state update protocols (e.g. averaging) within this article, and we do not derive the theoretical bounds for the rendezvous time of a buffered gossip algorithm; however, it is possible that current work in the literature on the voter model and Label Propagation Algorithm may be useful for future research that examines this particular issue.

### An Analytical Framework for Buffered Gossip Algorithms

To study of the behavior of the network as a whole, we must first understand the behavior of the individual nodes. A node that uses a buffered gossip algorithm performs three basic actions: *update state*, *transmit state*, and *erase buffer*. When the internal clock of node 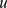 ticks, the first thing that node 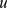 does is to update its state value according to a state update protocol. Next, the updated state value is transmitted to and stored in the buffer of a randomly chosen neighbor. After transmission has occurred, node 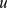 clears its buffer and awaits a new set of transmissions. This process of updating state, transmitting from node 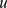 to a neighbor 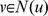, and buffer erasing is described by the following action algorithm:

1. **procedure** ACT(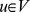, 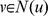)

2. 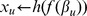


3. 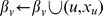


4. 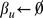


5. **end procedure**

When every node in a network uses a buffered gossip algorithm, an *adoption matrix*, denoted 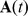, can be used to represent the spread of information at the end of the 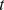
th time step. Let 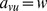 denote an element in the adoption matrix. The value 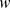 represents the how much of 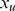 is used by node 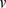 when determining 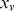. Consequently, 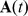 defines a weighted graph of 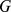 in which 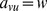 indicates an edge from node 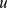 to node 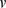 with weight 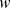.

Adoption matrices are constructed by a *network level* state update protocol of the form 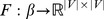, where 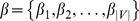. Network level state update protocols are algorithms that simplify the analysis of an entire network by creating adoption matrices from the buffers within of all nodes within a network. In the discrete domain, we want adoption matrices to be row stochastic so that they satisfy the conditions 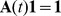 and 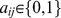. If an adoption matrix does not satisfy these conditions, then it reflects one or more illogical state updates (e.g. an agent attempts to be in two unique places at once, or partially present in multiple locations). We can construct a row stochastic adoption matrix using the network level state update protocol 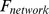, where 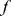 is the selection-based state update protocol of an individual node (e.g. proportional selection or maximum frequency selection) and 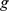 is the node selection companion function.

1. **function** 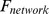
(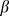)

2. 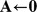


3. **for all** 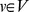 **do**

4. 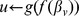


5. 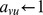


6. **end for**

7. **if** 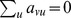 **then**

8. 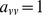


9. **end if**

10.**return** 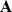


11. **end function**

Once an adoption matrix has been constructed, the rows indicate which state values node 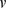 used to determine 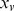 at the end of the *t*th time step and the columns indicate which nodes received 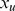 at the end of the *t*th time step. For example, if 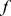 is proportional selection, then for each node 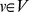, 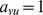 where node 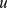 is chosen uniformly from 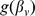. Similarly, if 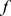 is maximum frequency selection, then for each node 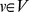, 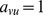 where node 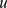 is chosen such that it is associated with the most frequently occurring state value present in 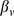 (i.e. 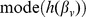).

Using these adoption matrices, we can study how the distribution of state values changes over time within a network of nodes that all use a buffered gossip algorithm. We can model these changes as the evolution of the linear system 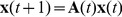
(1)where 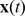 is the state vector of the nodes at the end of the 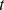
th time step. Under these dynamics, the decentralized rendezvous problem is solved when 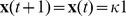, where 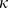 is the *consensus state* of the system.

### Convergence to a Consensus State

The first step in showing that a buffered gossip algorithm is capable of solving the decentralized rendezvous problem is to identify the conditions under which a consensus will form within a network of nodes using the algorithm. When using a buffered gossip algorithm, this can occur as the result of an *information cascade*: when the state value of a root node is propagated to every other node in the network. A *consensus sequence* specifies an ordered sequence of adoptions that cause an information cascade.

**Definition 1** *A consensus sequence is a finite set* 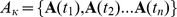 *with* 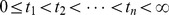 *such that* 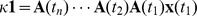 *. A consensus sequence specifies an ordered sequence of adoptions that propagate a single value to every node in the network.*

For any specific network, there may be multiple consensus sequences. Semantically, each matrix in a consensus sequence can be associated with an adjacency matrix that represents a path in 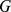. These paths denote the flow of information between nodes at the end of the associated time step.

We will now show that if a finite network, 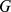, contains a directed spanning tree and if the nodes in 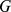 use a buffered gossip algorithm with a selection-based state update protocol and an asynchronous timing model, then at least one consensus sequence exists (lemma 1) and state information will eventually be transmitted according to that sequence (lemma 2).

**Lemma 1** *If a finite network,* 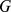 *, has a directed spanning tree and if the nodes in* 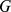 *use a buffered gossip algorithm with a selection-based state update protocol and an asynchronous timing model, then a consensus sequence exists.*

**Proof 1** *The proof of lemma 1 is similar to a breadth first search.*

*Let* 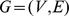 *be a finite network, let* 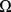 *be a directed spanning tree of* 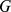 *with root* 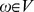 *, and let* 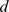 *be the number of nodes that will act during time step* 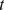.

*Because* 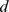 *is Poisson distributed when an asynchronous timing model is used,* 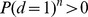 *if* 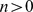 *is finite (i.e. there is a positive probability that only one node will be active* 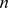 *times in a row). Because* 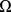 *is a directed spanning tree of* 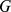 *with root* 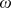 *,* 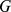 *is connected and there is at least one path from* 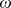 *to every other node in the network. Because nodes act independently and* 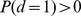 *,* 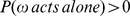 *at some time step* 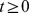 *(i.e. it is possible for* 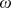 *to be the only node to act during an arbitrary time step). Likewise, if the* 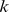 *children of* 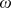 *are enumerated as* 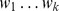 *, then* 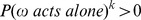 *for some* 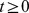 *. Because all nodes use a buffered gossip algorithm, each time* 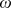 *acts it will transmit to one and only one neighbor. Because neighbors are selected uniformly at random, if* 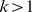 *there is a positive probability that the selected neighbor will not have already received* 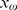 *in the previous* 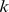 *time steps. Thus, after* 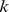 *time steps,* 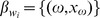 *for the* 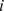 *th child of* 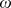 *(i.e. it is possible for* 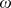 *to sequentially transmit its state value to each child, one after the other).*

*Similarly, there is a positive probability that after* 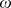 *has executed* 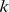 *transmissions, each child of* 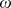 *,* 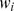 *, will act* 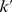 *time steps in a row and pass along* 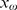 *to their* 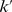 *children, because node* 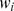 *will adopt* 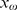 *as their own state since all nodes use a selection-based state update protocol and* 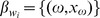 *. This process will continue recursively until* 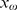 *has been adopted by every node in the network, one level at a time, moving from root to leaf.*

*There are* 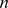 *adoption matrices corresponding to all of these single-node actions.* 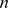 *is finite because* 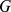 *is finite. Thus, the finite set of these matrices form one possible consensus sequence.*

**Figure 1:**
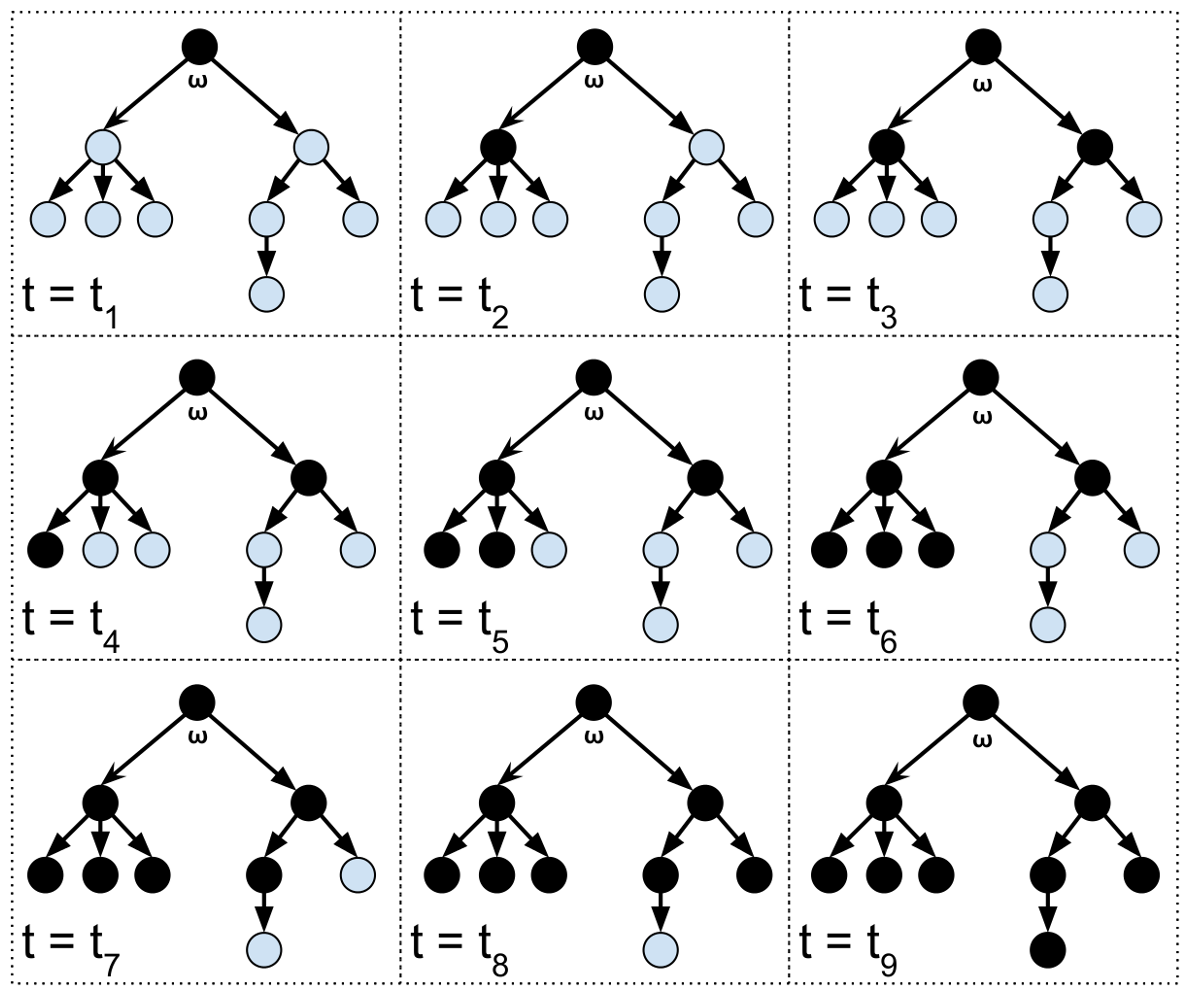
Transmission of information along the nodes of a directed spanning tree, 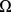, with root node, 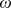.

Figure 1 visualizes the transmission process along 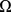 that is described in the proof of lemma 1. Given a network, 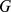, that has a directed spanning tree, 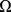, the root node, 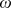, starts out in state black and proceeds to transmit that information to its children over the next two ticks. Those child then pass along the black information to their children over the next five ticks. Finally, consensus is achieved when the last node adopts the black information during the ninth tick.

**Lemma 2** *If a consensus sequence exists, then it will occur in asymptotic time with probability* 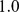.

**Proof 2** *Let* 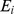 *be the event “A consensus sequence is observed during the time period* 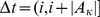 *.”* 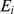 *is independent from* 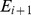 *because each node acts independently of one another and of past histories. Furthermore,* 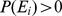 *for all* 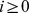 *because lemma 1 establishes the existence of* 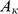 *. Thus,* 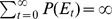 *. Hence, by the second Borel-Cantelli Lemma, the number of observations of* 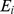 *will approach infinity as* 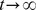 *and so the probability of observing a consensus sequence in asymptotic time is* 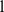 *.*

Combining lemma 1 and lemma 2, we can now state the criteria for consensus under a buffered gossip algorithm.

**Theorem 3** *If a finite network,* 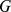 *, contains a directed spanning tree and if the nodes in* 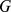 *use a buffered gossip algorithm with a selection-based state update protocol and an asynchronous timing model, then consensus will be obtained in asymptotic time.*

**Proof 3** *By direct application of lemma 1 and lemma 2.*

It should be noted that, in practice, a consensus sequence does not always reflect a tree. Initial configurations and simultaneous action can lead to actual consensus sequences that are much shorter than one might expect based on the naive sequences constructed in lemma 1.

### The Impact of Noise and Node Failure

The second step in showing that a buffered gossip algorithm is capable of solving the decentralized rendezvous problem is to show that it is robust to noise and node failure. Noise occurs when, for whatever reason, incorrect information is either transmitted or received. Node failure occurs when a node stops transmitting.

Based on theorem 3, we can conclude that noise will not prevent consensus, but it may interfere with the formation of a consensus sequence and thus reduce the speed at which consensus occurs. Because the information transmitted between nodes may not be accurate in the presence of noise, partially formed consensus sequences may be broken. However, because noise is random, there is a positive probability that a consensus sequence is able to form without disruption, and so lemma 1 and lemma 2 continue to hold. One interesting consequence of the buffered gossip algorithm's robustness to noise is that even though consensus will be obtained, it is possible that the final consensus state is an error value. Typically, this is undesirable behavior - but it could be leveraged by intelligent social agents as the basis of creativity, exploration, and innovation.

We can also conclude from theorem 3 that node failure will only prevent consensus when two conditions hold: 1) the node(s) that fail are cut points within every possible directed spanning tree of 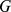; i.e. their removal results in the inability to construct a directed spanning tree in 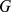; and 2) the node(s) that fail never reactivate. If both of these conditions do not hold, then node failure will only delay the formation of a consensus by the same argument given on the impact of noise.

These conclusions align with the existing knowledge that robustness to noise and node failure is one of the major strengths of a gossip-based approach to consensus formation [1], [2], [9].

### Stability of the Consensus State

The final step in showing that a buffered gossip algorithm is capable of solving the decentralized rendezvous problem is to show that once a consensus has been obtained, the consensus will be maintained until new information becomes available. Theorem 3 establishes that buffered gossip algorithms are capable of solving the decentralized rendezvous problem by achieving consensus in the context of locational information, but it does not ensure that the system will maintain that consensus once it has been obtained.

Lemma 4 ensures that if the system achieves consensus, it will remain in consensus until acted upon by external forces.

**Lemma 4** *If a finite network,* 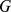 *, contains a directed spanning tree and if the nodes in* 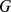 *use a buffered gossip algorithm with a selection-based state update protocol and an asynchronous timing model, then* 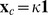 *is a fixed point of* 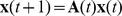 *.*

**Proof 4** *By construction,* 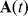 *is row stochastic, so* 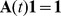 *. Thus,* 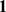 *is an eigenvector of* 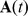 *with an eigenvalue of* 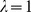 *. Because scalar multiples of eigenvectors are also eigenvectors,* 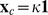 *is an eigenvector of* 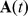 *with an eigenvalue* 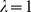 *. So* 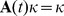 *, and thus the consensus state,* 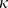 *, is a fixed point of* 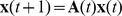 *.*

Thus, a buffered gossip algorithm with a selection-based state update protocol and an asynchronous timing model is a solution to the decentralized rendezvous problem in the discrete domain with direct communication if the finite network, 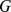, contains a directed spanning tree.

## The Uniform Gossip Algorithm as an Alternative Solution to the Rendezvous Problem

We have described the buffered gossip algorithm and shown that it is capable of solving the decentralized rendezvous problem in a discrete domain with direct communication. We now present the uniform gossip algorithm [9], [10], [24] as a buffered gossip algorithm with a very special state update protocol and show that it too is capable of solving the decentralized rendezvous problem in a discrete domain with direct communication.

### The Uniform Gossip Algorithm as a Randomized Gossip Process

In the original description of the uniform gossip algorithm [9], nodes transmit their state value to a neighbor that has been selected according to a uniform distribution, and that neighbor then immediately updates its own state value to reflect the newly received information. As a result of this process, the state value of each node at the end of a time step reflects the last transmission that it received.

We can encapsulate the behavior of the uniform gossip algorithm as a buffered gossip algorithm in which the buffer is ordered by transmission sequence and *tail selection* is used as the state update protocol. Tail selection, denoted 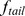, is a selection-based state update protocol that selects the last element in the buffer. For example, if 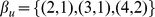 then 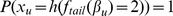. Nodes using tail selection are equivalent to nodes that lack a buffer for long-term storage and overwrite their state value in response to every transmission. As such, the uniform gossip algorithm is only affected by the randomness of the incoming transmissions. The uniform gossip algorithm uses the same gossip mechanism and gossip protocol as the buffered gossip algorithm. Like a buffered gossip algorithm with proportional selection, the uniform gossip algorithm is similar to a voter model on a network with a time-varying topology. At any given time step, 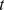, the neighborhood of each node, 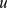, consists only of those nodes transmitting to node 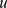.

Because the uniform gossip algorithm is an established algorithm for information dissemination and because it can be framed as a buffered gossip algorithm, it offers an ideal point of comparison for evaluating the effectiveness of other buffered gossip algorithms such as those using a proportional selection or maximum-frequency selection state update protocol.

### The Uniform Gossip Algorithm as a Solution to the Rendezvous Problem

The uniform gossip algorithm is guaranteed to solve the decentralized rendezvous problem under the same conditions as the buffered gossip algorithm.

According to theorem 3, if a finite network, 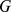, contains a directed spanning tree and if the nodes in 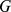 use a buffered gossip algorithm with a selection-based state update protocol and an asynchronous timing model, then consensus will be obtained in asymptotic time. The uniform gossip algorithm can be modeled as a buffered gossip algorithm with tail selection. Tail selection is a selection-based state update protocol. Therefore, if a finite network, 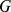, contains a directed spanning tree and if the nodes in 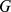 use the uniform gossip algorithm with an asynchronous timing model, then consensus will be obtained in asymptotic time. In the context of the decentralized rendezvous problem in the discrete domain with direct communication, consensus is obtained on a rendezvous location.

By the same logic, the uniform gossip algorithm has the same robustness to noise and node failure as other buffered gossip algorithms with selection-based state update protocols, and lemma 4 provides the conditions under which a consensus formed by the uniform gossip algorithm is stable.

Thus, the uniform gossip algorithm with an asynchronous timing model is a solution to the decentralized rendezvous problem in the discrete domain with direct communication if the finite network, 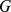, contains a directed spanning tree.

## Application to Discrete Rendezvous

We have introduced the buffered gossip algorithm and described the uniform gossip algorithm as a randomized gossip process and have shown that both of these algorithms are theoretically capable of solving the decentralized rendezvous problem in the discrete domain with direct communication. Now, we use a multi-agent simulation to verify that these algorithms are capable of solving it in practice, and to compare their speed relative to one another. We restrict our current focus to buffered gossip algorithms with proportional selection and maximum frequency selection state update protocols because they are similar to existing techniques of information propagation (the voter model and the label propagation algorithm); although because they are being used in a new context we cannot be guaranteed that they will display the same behavior. We also make the simplifying assumption that in the event of a node receiving multiple transmissions from the same neighbor prior to a state update, only the most recent transmission is kept in the buffer. Finally, because we are focused on comparing rendezvous speed between different algorithms, and because we have previously shown that noise and node failure only prevent consensus formation in very specific scenarios, we assume here that information is transmitted without error and nodes do not fail during consensus formation. This assumption allows us to simplify our experiments by holding the noise and node failure probabilities at 0.0.

To compare the relative rendezvous speed of buffered gossip algorithm and the uniform gossip algorithm, we test the following hypotheses:

- Because maximum frequency selection is explicitly designed to be less random than proportional selection, we expect that the mean rendezvous time of a buffered gossip algorithm using maximum frequency selection (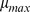) is less than the the mean rendezvous time of a buffered gossip algorithm using proportional selection (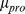). Supporting evidence for this expectation exists if we are able to reject the null hypothesis: 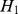: 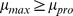.
- Because maximum frequency selection is explicitly designed to be less random than the uniform gossip algorithm, we expect that the mean rendezvous time of a buffered gossip algorithm using maximum frequency selection (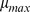) is less than the the mean rendezvous time of the uniform gossip algorithm (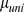). Supporting evidence for this expectation exists if we are able to reject the null hypothesis: 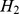: 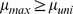.
- Because randomness is a core component of proportional selection and the uniform gossip algorithm, we expect that the mean rendezvous time of a buffered gossip algorithm using proportional selection (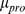) is equal to the the mean rendezvous time of the uniform gossip algorithm (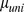). Supporting evidence for this expectation exists if we fail to reject the null hypothesis: 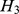: 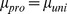.

We also consider the impact of network topology on rendezvous speed by testing hypotheses related to four different types of networks (random, lattice, scale-free, and small world):

- Because differences in network topology have been found to affect the performance of the label propagation algorithm [35], and because the label propagation algorithm is the basis for maximum frequency selection, we expect that there will be differences between the mean rendezvous times of a buffered gossip algorithm using maximum frequency selection on a random network (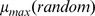), a scale-free network (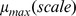), a small world network(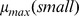), and a lattice network (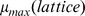). Supporting evidence for this expectation exists if we are able to reject the null hypothesis: 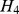: 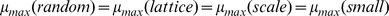.
- Because differences in network topology have been found to affect the performance of the voter model [37], and because the voter model is the basis for proportional selection, we expect that there will be differences between the mean rendezvous times of a buffered gossip algorithm using proportional selection on a random network (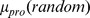), a scale-free network (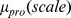), a small world network(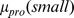), and a lattice network (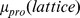). Supporting evidence for this expectation exists if we are able to reject the null hypothesis: 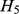: 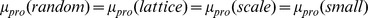.
- Because differences in network topology have been found to affect at least one randomized algorithm used in information propagation (e.g. the voter model [37]), and because randomness is a core component of the uniform gossip algorithm, we expect that there will be differences between the mean rendezvous times of the uniform gossip algorithm on a random network (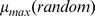), a scale-free network (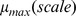), a small world network(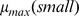), and a lattice network (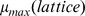). Supporting evidence for this expectation exists if we are able to reject the null hypothesis: 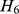: 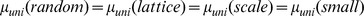.

Finally, given the established literature that illustrates the potential impacts of network topology, we expect that the relative performance of a buffered gossip algorithm using proportional selection or maximum frequency selection, and the uniform gossip algorithm, differs across network topologies. For consistency, we denote this test as 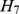 and verify it by graphical analysis.

### Experimental Design

We use a multi-agent simulation, written in Python with the NetworkX [39] and Numpy [40] libraries, to conduct a series of simulation experiments to gather data relevant to the performance of a buffered gossip algorithm using proportional selection or maximum frequency selection, and the uniform gossip algorithm using tail selection.

We consider a scenario in which a population of 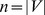 agents, connected by a static communication network, act as an autonomous, decentralized, multi-agent surveillance system with an asynchronous timing model. Each agent in this system is responsible for monitoring a portion of the overall surveillance area, and each agent is as structurally and computationally as simple as possible to accomplish this task. As a consequence, no one agent can maintain an accurate picture of the entire environment. In order to construct an accurate picture of the environment, the population must periodically rendezvous at one of 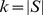 relay hubs in order to sequence and transmit the individual information pieces back to a remote storage location. If any individual agent does not rendezvous, then the information sent back for analysis and storage is incomplete.

We simplify this scenario by assuming that the communication network between agents is static. Each node in the network represents an agent and an edge connects two nodes if there is a communication link between the associated agents. The state value of each node represents that node's desired rendezvous location and is encoded as an integer value. Each node can store up to 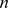 transmissions in its buffer, and those transmissions are stored in the order in which they are received. If a node receives multiple transmissions from the same agent before it is able to clear its buffer, only the most recent transmission is retained. To test hypotheses 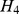, 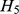, and 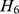, we allow the communication network to be either an Erdös-Renyi random network, a Barabasi-Albert scale-free network, a Newman-Watts-Strogatz small world network [41], or a lattice network.

Nodes use an asynchronous timing model, where the expected number of nodes that act in a single time step follows a Poisson distribution with 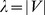. Because asynchronous timing models are used, it is possible that some nodes will act multiple times within a single time step. Simulation time is measured in *steps*. One step has passed when all active nodes have updated their state value and spread their information in accordance with their action algorithm. Thus, one step is equivalent to one time step. Those nodes that act within a single step do so in a uniformly random order.

The state update protocol (proportional selection, maximum frequency selection, or tail selection) and the network topology (Erdös-Renyi random, Barabasi-Albert scale-free, Newman-Watts-Strogatz small world, or lattice) are the primary independent variables. For each combination of state update protocol and network topology, we randomly construct 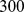 networks with the selected topological structure and then conduct 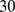 independent simulations of rendezvous over each network. These networks are constructed randomly, with 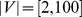 and 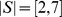 being chosen according to a uniform distribution. The decision to vary network and state space size was made to test solution potential over a wide range of possibilities. Additionally, Erdös-Renyi random networks use a random value in the range 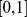 for their connection probability, and are guaranteed to be connected; Barabase-Albert scale-free networks and Newman-Watts-Strogatz small world networks are randomly parameterized based on the number of nodes in the network; and lattice networks are guaranteed to be square and do not wrap to form a torus.

The consensus time (measured in steps) is the dependent variable under study, with the characterization that a value of 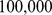 represents a failure to achieve consensus. Nodes successfully rendezvous if the state of every node is identical within 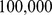 steps. Nodes fail to rendezvous if either periodic behavior is observed or the simulation runs in excess of a maximum time limit (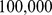 steps). The simulation software is capable of detecting periodic behavior of up to 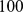 unique states. Behavior is considered to be periodic if a sequence of state distributions repeats continuously for 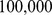 consecutive steps (e.g. a sequence of 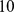 state distributions repeats 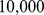 times in a row).

Each simulation runs until either consensus is reached, a non-consensus stable state is observed (either fixed or periodic), or a time limit of 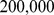 steps is exceeded. This produces a total of 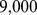 data points per experimental configuration. To remove randomness as a cause for differences between experimental configurations, each configuration is initialized with same sequence of random numbers (i.e. simulation 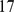 of the configuration {proportional, random} uses the same random seed as simulation 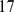 of the configuration {maximum, lattice}).

### Experimental Results

Having described our hypotheses and experimental design, we now discuss the results of our experiments 300 randomly generated Erdös-Renyi random networks, 300 randomly generated Barabasi-Albert scale-free networks, 300 randomly generated Newman-Watts-Strogatz small world networks, and 300 randomly generated lattice networks are discussed below.

#### Erdös-Renyi Random Networks

**Figure 2:**
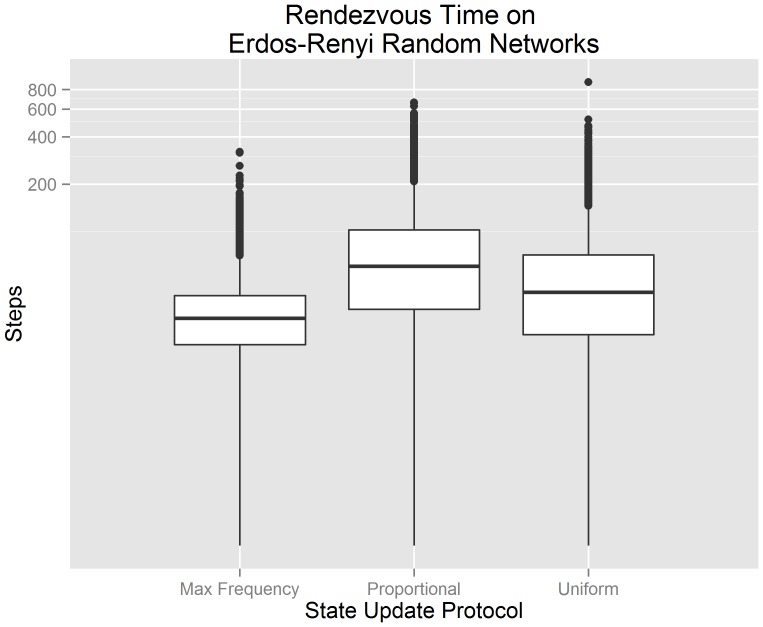
Box plots for the rendezvous time on Erdös-Renyi random networks showing the interquartile range, median value, and outliers. It can be observed that buffered gossip algorithms using maximum frequency selection generally have a lower rendezvous time than the tested alternatives.

Figure 2 visualizes our experimental data from 300 randomly generated Erdös-Renyi random networks using a standard box plot. The upper and lower boundaries of each box correspond to the first and third quartile of the data, with the middle line represents the median value. The upper and lower whiskers extend out to the largest and smallest value within 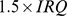 of the boundary. The individual points represent the outliers of the observed data. The x-axis indicates the state update protocol used by each algorithm. The y-axis indicates the number of steps until consensus is achieved. The y-axis has been transformed logarithmically in order to improve the overall visualization of the data; the data itself has not been transformed. We observe that a buffered gossip algorithm using maximum frequency selection has the lowest median rendezvous time and smallest third quartile of the three algorithms. These observations suggest that, when agents communicate over Erdös-Renyi random networks, a buffered gossip algorithm using maximum frequency selection should produce lower rendezvous times in comparison to a buffered gossip algorithm using proportional selection or the uniform gossip algorithm.

**Figure 3:**
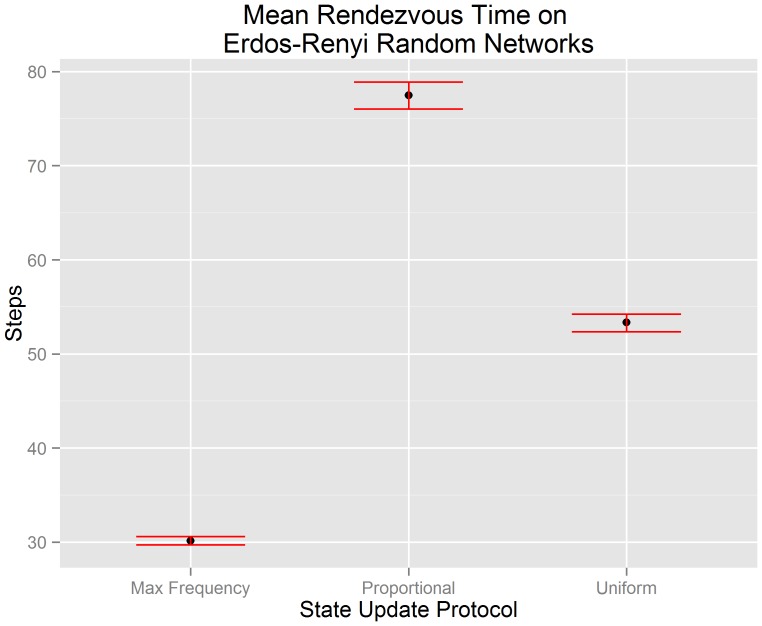
Errorbar plots of the 95% confidence intervals for the mean rendezvous time on Erdös-Renyi random networks. It can be observed that a buffered gossip algorithm using maximum frequency selection has a mean rendezvous time less than a buffered gossip algorithm using proportional selection or the uniform gossip algorithm. Furthermore, the mean rendezvous time of a buffered gossip algorithm using proportional selection is not equal to the mean rendezvous time of the uniform gossip algorithm.

Figure 3 visualizes the mean rendezvous time of our random network data along with the 95% confidence interval of each mean. The x-axis indicates the state update protocol used by each algorithm. The y-axis indicates the number of steps until consensus is achieved. We test hypotheses 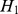 (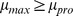), 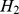 (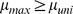), and 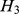 (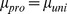) in the context of Erdös-Renyi random networks using the data visualized in Figure 3. We reject hypotheses 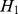 and 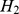 (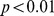 for both). This suggests that there is evidence to support the claim that the mean rendezvous time of a buffered gossip algorithm using maximum frequency selection (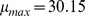) is less than the the mean rendezvous time of a buffered gossip algorithm using proportional selection (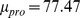) and less than the mean rendezvous time of the uniform gossip algorithm (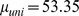). We also reject 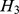 (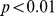), and so there is not evidence to support the claim that the mean rendezvous time of a buffered gossip algorithm using proportional selection (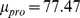) is equal to the the mean rendezvous time of the uniform gossip algorithm (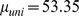). Instead, the evidence suggests that the mean rendezvous time of the uniform gossip algorithm is less than the mean rendezvous time of a buffered gossip algorithm using proportional selection. The rejection of 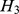 may suggest that even though randomness is central to proportional selection and the uniform gossip algorithm, there are other factors that we have not yet examined that may influence the length of time required to rendezvous.

#### Barabasi-Albert Scale-Free Networks

**Figure 4:**
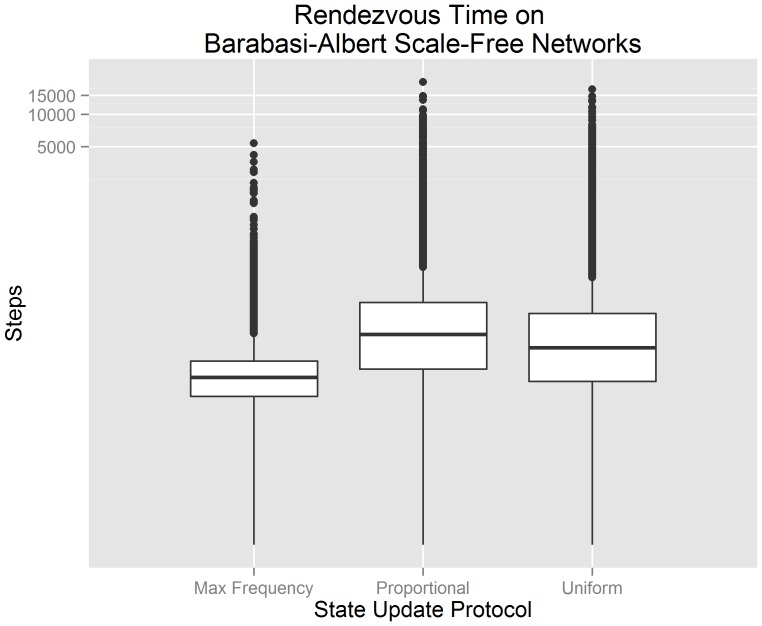
Box plots for the rendezvous time on Barabasi-Albert scale-free networks showing the interquartile range, median value, and outliers. It can be observed that buffered gossip algorithms using maximum frequency selection generally have a lower rendezvous time than the tested alternatives.

Figure 4 visualizes our experimental data from 300 randomly generated Barabasi-Albert scale-free networks using a standard box plot. The x-axis indicates the state update protocol used by each algorithm. The y-axis indicates the number of steps until consensus is achieved. The y-axis has been transformed logarithmically in order to improve the overall visualization of the data; the data itself has not been transformed. We observe that a buffered gossip algorithm using maximum frequency selection has the lowest median rendezvous time and smallest third quartile. These observations suggest that, when agents communicate over Barabasi-Albert scale-free networks, a buffered gossip algorithm using maximum frequency selection should produce lower rendezvous times in comparison to a buffered gossip algorithm using proportional selection or the uniform gossip algorithm.

**Figure 5:**
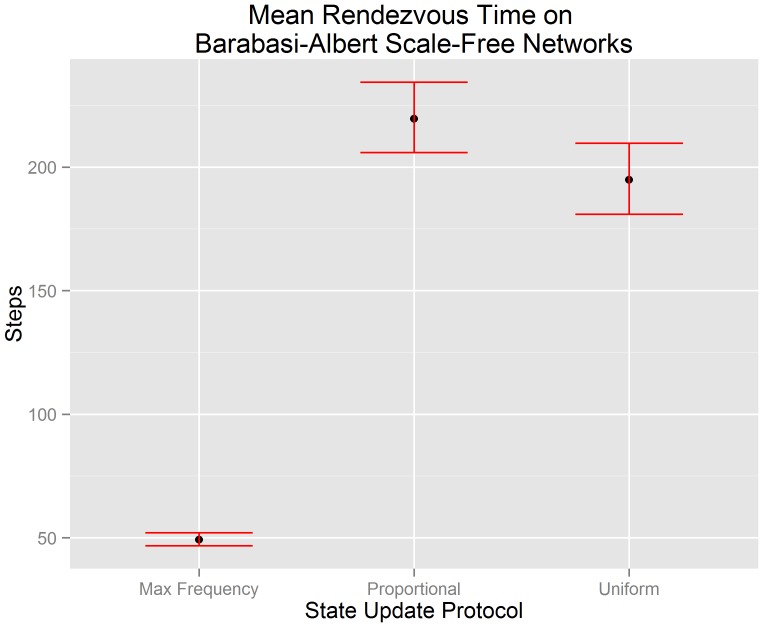
Errorbar plots of the 95% confidence intervals for the mean rendezvous time on Barabasi-Albert scale-free networks. It can be observed that a buffered gossip algorithm using maximum frequency selection has a mean rendezvous time less than a buffered gossip algorithm using proportional selection or the uniform gossip algorithm. Furthermore, the mean rendezvous time of a buffered gossip algorithm using proportional selection is not equal to the mean rendezvous time of the uniform gossip algorithm, but it is close.

Figure 5 visualizes the mean rendezvous time of our scale-free network data along with the 95% confidence interval of each mean. The x-axis indicates the state update protocol used by each algorithm. The y-axis indicates the number of steps until consensus is achieved. We test hypotheses 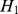 (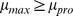), 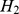 (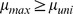), and 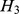 (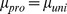) in the context of scale-free networks using the experimental data that underlies Figure 5. We reject hypotheses 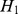 and 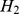 (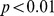 for both). This suggests that there is evidence to support the claim that the mean rendezvous time of a buffered gossip algorithm using maximum frequency selection (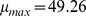) is less than the the mean rendezvous time of a buffered gossip algorithm using proportional selection (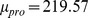) and less than the mean rendezvous time of the uniform gossip algorithm (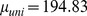). We also reject 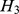 (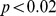), and so there is not sufficient evidence to support the claim that the mean rendezvous time of a buffered gossip algorithm using proportional selection (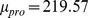) is equal to the the mean rendezvous time of the uniform gossip algorithm (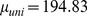). Instead, the evidence suggests that the mean rendezvous time of the uniform gossip algorithm is less than the mean rendezvous time of a buffered gossip algorithm using proportional selection. The rejection of 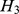 may suggest that even though randomness is central to proportional selection and the uniform gossip algorithm, there are other factors that we have not yet examined that may influence the length of time required to rendezvous.

#### Newman-Watts-Strogatz Small World Networks

**Figure 6:**
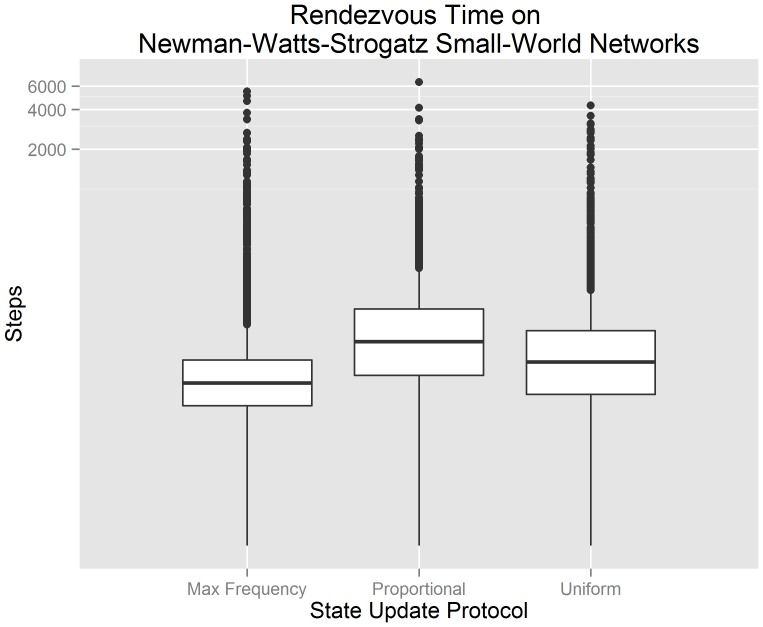
Box plots for the rendezvous time on Newmann-Watts-Strogatz small world networks showing the interquartile range, median value, and outliers. It can be observed that buffered gossip algorithms using maximum frequency selection generally have a lower rendezvous time than the tested alternatives.

Figure 6 visualizes our experimental data from 300 randomly generated Newman-Watts-Strogatz small world networks using a standard box plot. The x-axis indicates the state update protocol used by each algorithm. The y-axis indicates the number of steps until consensus is achieved. The y-axis has been transformed logarithmically in order to improve the overall visualization of the data; the data itself has not been transformed. We observe that a buffered gossip algorithm using maximum frequency selection has the lowest median rendezvous time and smallest third quartile. These observations suggest that, when agents communicate over Newman-Watts-Strogatz small world networks, a buffered gossip algorithm using maximum frequency selection should produce lower rendezvous times in comparison to a buffered gossip algorithm using proportional selection or the uniform gossip algorithm.

**Figure 7:**
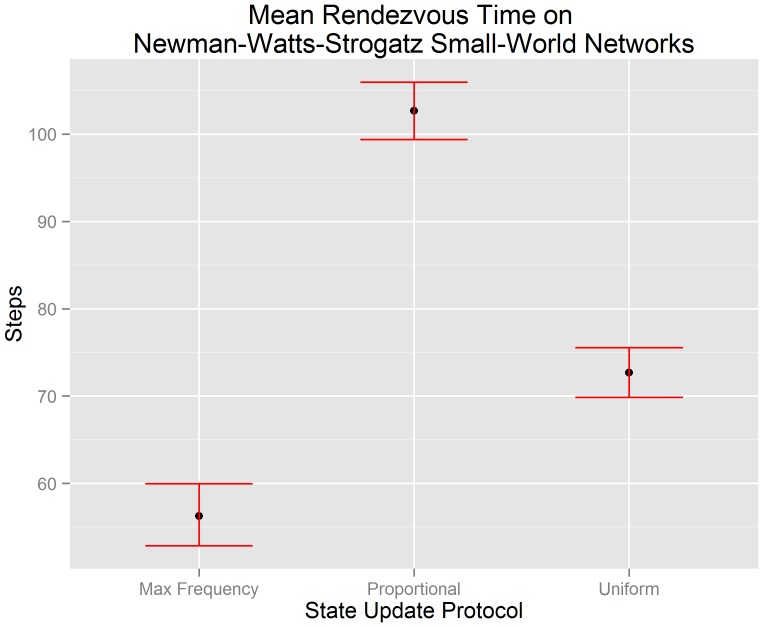
Errorbar plots of the 95% confidence intervals for the mean rendezvous time on Newmann-Watts-Strogatz small world networks. It can be observed that a buffered gossip algorithm using maximum frequency selection has a mean rendezvous time less than a buffered gossip algorithm using proportional selection or the uniform gossip algorithm. Furthermore, the mean rendezvous time of a buffered gossip algorithm using proportional selection is not equal to the mean rendezvous time of the uniform gossip algorithm; it is greater.

Figure 7 visualizes the mean rendezvous time of our small world network data along with the 95% confidence interval of each mean. The x-axis indicates the state update protocol used by each algorithm. The y-axis indicates the number of steps until consensus is achieved. We test hypotheses 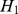 (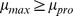), 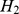 (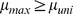), and 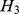 (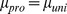) in the context of small world networks using the experimental data that underlies Figure 7. We reject hypotheses 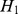 and 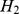 (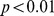 for both). This suggests that there is evidence to support the claim that the mean rendezvous time of a buffered gossip algorithm using maximum frequency selection (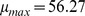) is less than the the mean rendezvous time of a buffered gossip algorithm using proportional selection (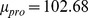) and less than the mean rendezvous time of the uniform gossip algorithm (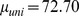). We also reject 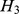 (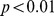), and so there is not evidence to support the claim that the mean rendezvous time of a buffered gossip algorithm using proportional selection (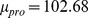) is equal to the the mean rendezvous time of the uniform gossip algorithm (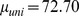). Instead, the evidence suggests that the mean rendezvous time of the uniform gossip algorithm is less than the mean rendezvous time of a buffered gossip algorithm using proportional selection. The rejection of 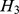 may suggest that even though randomness is central to proportional selection and the uniform gossip algorithm, there are other factors that we have not yet examined that may influence the length of time required to rendezvous.

#### Lattice Networks

**Figure 8:**
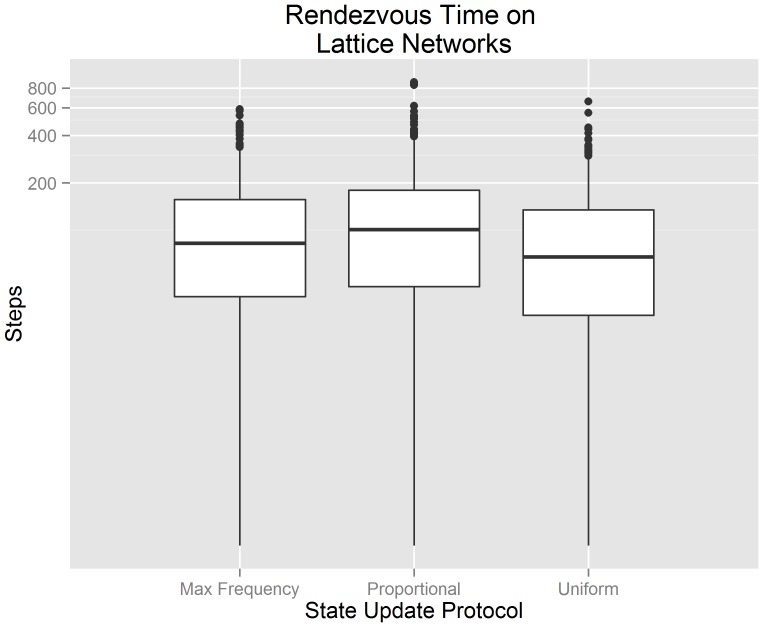
Box plots for the rendezvous time on lattice networks showing the interquartile range, median value, and outliers. It can be observed that the rendezvous times between the buffered and uniform gossip algorithms fall within relatively the same range. This suggests that overall performance is similar among the tested solutions.

Figure 8 visualizes our experimental data from 300 randomly generated lattice networks using a standard box plot. The x-axis indicates the state update protocol used by each algorithm. The y-axis indicates the number of steps until consensus is achieved. The y-axis has been transformed logarithmically in order to improve the overall visualization of the data; the data itself has not been transformed. We observe that the uniform gossip algorithm has the lowest median rendezvous time and smallest third quartile. We also observe that the total performance range (including outliers) is similar between a buffered gossip algorithm using maximum frequency selection, a buffered gossip algorithm using proportional selection and the uniform gossip algorithm; although the median value and third quartile of a buffered gossip algorithm using maximum frequency selection is less than the median value and third quartile of the proportional data. These observations suggest that when agents communicate through lattice random networks, the uniform gossip algorithm should produce lower rendezvous times in comparison to a buffered gossip algorithm using maximum frequency selection or proportional selection, but it would not be uncommon for all three algorithms to produce similar results.

**Figure 9:**
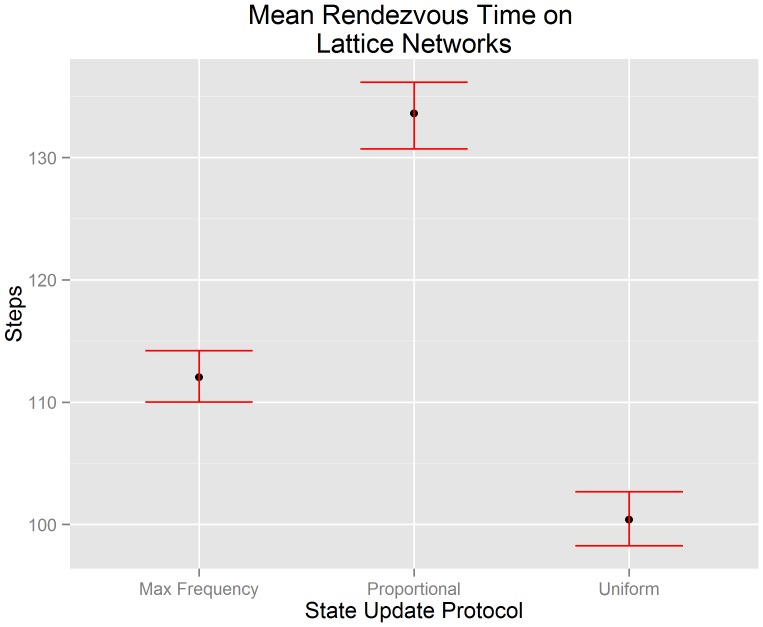
Errorbar plots of the 95% confidence intervals for the mean rendezvous time on lattice networks. It can be observed that the uniform gossip algorithm has a mean rendezvous time less than a buffered gossip algorithm using maximum frequency selection or proportional selection. Furthermore, the mean rendezvous time of a buffered gossip algorithm using proportional selection is not equal to the mean rendezvous time of the uniform gossip algorithm.

Figure 9 visualizes the mean rendezvous time of our lattice network data along with the 95% confidence interval of each mean. The x-axis indicates the state update protocol used by each algorithm. The y-axis indicates the number of steps until consensus is achieved. We test hypotheses 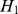 (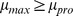), 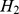 (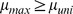), and 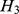 (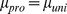) in the context of lattice networks using the experimental data that underlies Figure 9. We reject hypotheses 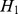 (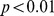). This suggests that there is evidence to support the claim that the mean rendezvous time of a buffered gossip algorithm using maximum frequency selection (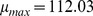) is less than the the mean rendezvous time of a buffered gossip algorithm using proportional selection (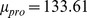). We fail to reject 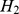 (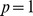). This suggests that there is not evidence to support the claim that the mean rendezvous time of a buffered gossip algorithm using maximum frequency selection (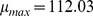) is less than the mean rendezvous time of the uniform gossip algorithm (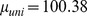). We also reject 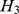 (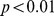), and so there is also not evidence to support the claim that the mean rendezvous time of a buffered gossip algorithm using proportional selection (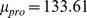) is equal to the the mean rendezvous time of the uniform gossip algorithm (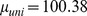). Instead, as in a random network, the evidence suggests that the mean rendezvous time of the uniform gossip algorithm is less than the mean rendezvous time of a buffered gossip algorithm using proportional selection. The rejection of 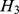 may suggest that even though randomness is central to proportional selection and the uniform gossip algorithm, there are other factors that we have not yet examined that may influence the length of time required to rendezvous.

### The Impact of Network Topology

In regards to comparing the rendezvous time across network topology, we reject 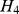, 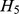, and 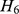 (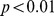 for each). The evidence supports the claim that rendezvous time is sensitive to the topology of the agent communication network. Furthermore, we observe evidence to support hypothesis 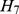. The communication topology appears to produce a difference in the relative performance of a buffered gossip algorithm proportional selection or maximum frequency selection, and the uniform gossip algorithm.

### Results Summary

Rendezvous was observed in all of our experimental configurations. In the worst case, the maximum rendezvous time was 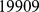 steps and occurred under proportional selection over a Barabasi-Albert scale-free network with 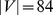 nodes, 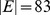 edges, and 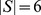.

Our results suggest that while the state update protocol does exhibit influence on the rendezvous time, the topology of the communication network may be the most critical factor in the speed of rendezvous. Evidence of this behavior is found in the observed rendezvous times across the four network topologies tested in our experiments. This finding is in line with the existing research on the voter model and Label Propagation Algorithm. Furthermore, the underlying theory of the buffered gossip algorithm also suggests that the topology is critical to the overall success of a rendezvous solution; e.g. disconnected networks will never achieve consensus.

Our results also suggest that a buffered gossip algorithm using maximum frequency selection may be preferable when the network topology is unknown, and perhaps even when it is time-dependent. In the worst case among our results (lattice networks), a buffered gossip algorithm using maximum frequency selection is only slightly worse than the uniform gossip algorithm. It is possible that other protocols, not examined in this current work, can provide even better performance; but, this is a topic for future studies.

## Conclusions

We introduce the buffered gossip algorithm as a solution to the decentralized rendezvous problem when there are a finite number of discrete meeting locations and there is direct communication between agents an their local neighbors. In addition, we frame the well known uniform gossip algorithm as a randomized gossip process. We show that when a buffered or uniform gossip algorithm is used with an asynchronous timing model and a selection-based state update protocol, rendezvous is guaranteed if the communication network between agents contains a directed spanning tree. Finally, we use a set of simulation experiments to compare the practical performance of the buffered gossip algorithm to the uniform gossip algorithm.

Our results indicate that both the buffered and uniform gossip algorithms offer an attractive solution to the decentralized rendezvous problem in a discrete domain where agents are able to directly communicate with one of their local neighbors. Buffered gossip algorithms ensure consensus in scenarios where agents act asynchronously and it is not possible (or not desired) for an agent to access, process, and re-transmit existing data prior to the introduction of new data. Buffered gossip algorithms also allow agents to use multiple information sources when determining their desired rendezvous location and allow agents to receive and decode multiple transmissions simultaneously. In our experiments, a buffered gossip algorithm using the maximum frequency selection protocol was able to reach consensus faster than the uniform gossip algorithm on three out of the four topologies that we examined. A buffered gossip algorithm using the maximum frequency selection protocol was always faster than a buffered gossip algorithm using the proportional selection protocol. The uniform gossip algorithm does not allow agents to use multiple information sources when deciding on their new state, but it is usable if a buffer is not desired or not feasible due to memory constraints, and in some cases (e.g. lattice networks) the uniform gossip algorithm is able to achieve a consensus on rendezvous location faster than a buffered gossip algorithm.

Generally, however, the choice of which gossip algorithm to use on the decentralized rendezvous problem in a discrete domain with direct communication appears to depend largely on the specific constraints of the problem, the state update protocol being considered, and the topology of the communication network between agents; but, the results of our study suggest that the best chance for success is likely to occur when a buffered gossip algorithm is used with the maximum frequency selection protocol.

By abstracting gossip algorithms into a framework that allows them to store multiple pieces of information in a buffer, the buffered gossip algorithm is able to take advantage of multiple information sources to make a more informed decision about the consensus state. Because the decentralized rendezvous problem is an instance of the consensus problem, our experimental results suggest that buffered gossip algorithms can replace many of the gossip algorithms currently being used in other problem domains that require consensus, such as leader election or norm emergence in open multi-agent systems [42].

## References

[1] Alon N, Barak A, Manber U (1987) On disseminating information reliably without broadcasting. In: ICDCS. IEEE Computer Society, pp. 74–81.

[2] Feige U, Peleg D, Raghavan P, Upfal E (1990) Randomized broadcast in networks. Random Structures & Algorithms 1: 447–460.

[3] Fang J, Morse A, Cao M (2008) Multi-agent rendezvousing with a finite set of candidate rendezvous points. In: American Control Conference, 2008. pp. 765–770. doi:10.1109/ACC.2008.4586585.

[4] Lin J, Morse A, Anderson BDO (2003) The multi-agent rendezvous problem. In: Decision and Control, 2003. Proceedings. 42nd IEEE Conference on. volume 2, pp. 1508–1513 Vol.2. doi: 10.1109/CDC.2003.1272825.

[5] Lin J, Morse A, Anderson BDO (2004) The multi-agent rendezvous problem - the asynchronous case. In: Decision and Control, 2004. CDC. 43rd IEEE Conference on. volume 2, pp. 1926–1931 Vol.2. doi:10.1109/CDC.2004.1430329.

[6] Olfati-Saber R, Fax J, Murray R (2007) Consensus and cooperation in networked multi-agent systems. Proceedings of the IEEE 95: 215–233.

[7] Pelc A (2011) Disc 2011 invited lecture: Deterministic rendezvous in networks: Survey of models and results. In: Peleg D, editor, Distributed Computing, Springer Berlin Heidelberg, volume 6950 of *Lecture Notes in Computer Science*. pp. 1–15.

[8] Ren W, Beard R, Atkins E (2007) Information consensus in multivehicle cooperative control. Control Systems, IEEE 27: 71–82.

[9] Karp R, Schindelhauer C, Shenker S, Vocking B (2000) Randomized rumor spreading. In: Proceedings 41st Annual Symposium on Foundations of Computer Science. IEEE, pp. 565–574. doi: 10.1109/SFCS.2000.892324.

[10] Kempe D, Kleinberg J, Demers A (2004) Spatial gossip and resource location protocols. Journal of the ACM 51: 943–967.

[11] Mosk-Aoyama D, Shah D (2006) Computing separable functions via gossip. In: Proceedings of the Twenty-fifth Annual ACM Symposium on Principles of Distributed Computing. New York, NY, USA: ACM, PODC ′06, pp. 113–122. doi:10.1145/1146381.1146401. URL http://doi.acm.org/10.1145/1146381.1146401.

[12] Lamport L (2001) Paxos made simple. ACM SIGACT News 32: 18–25.

[13] Liggett TM (2005) Interacting Particle Systems. Springer Berlin Heidelberg.

[14] Ren W, Beard R, Atkins E (2005) A survey of consensus problems in multi-agent coordination. In: Proceedings of the 2005, American Control Conference, 2005. IEEE, pp. 1859–1864.

[15] Dimakis AG, Kar S, Moura JM, Rabbat MG, Scaglione A (2010) Gossip Algorithms for Distributed Signal Processing. Proceedings of the IEEE 98: 1847–1864.

[16] Demers A, Greene D, Houser C, Irish W, Larson J, et al (1988) Epidemic algorithms for replicated database maintenance. ACM SIGOPS Operating Systems Review 22: 8–32.

[17] Hedetniemi SM, Hedetniemi ST, Liestman AL (1988) A survey of gossiping and broadcasting in communication networks. Networks 18: 319–349.

[18] Hromkovic J, Klasing R, Monien B, Peine R (1996) Dissemination Of Information In Interconnection Networks (Broadcasting & Gossiping). Combinatorial Network Theory: 125–212.

[19] Kempe D, Dobra A, Gehrke J (2003) Gossip-based computation of aggregate information. In: 44th Annual IEEE Symposium on Foundations of Computer Science, 2003. Proceedings. IEEE, pp. 482–491.

[20] Boyd S, Ghosh A, Prabhakar B, Shah D (2006) Randomized gossip algorithms. IEEE Transactions on Information Theory 52: 2508–2530.

[21] Kermarrec AM, van Steen M (2007) Gossiping in distributed systems. SIGOPS Oper Syst Rev 41: 2–7.

[22] Lamport L (1998) The part-time parliament. ACM Trans Comput Syst 16: 133–169.

[23] Ongaro D, Ousterhout J (2014) In search of an understandable consensus algorithm. In: Proceedings of the 2014 USENIX Conference on USENIX Annual Technical Conference. Berkeley, CA, USA: USENIX Association, USENIX ATC'14, pp. 305–320. URL http://dl.acm.org/citation.cfm?id=2643634.2643666.

[24] Kempe D, Kleinberg J (2002) Protocols and impossibility results for gossip-based communication mechanisms. In: The 43rd Annual IEEE Symposium on Foundations of Computer Science, 2002. Proceedings. IEEE Comput. Soc, pp. 471–480.

[25] Ganesh AJ, Kermarrec AM, Massoulie L (2003) Peer-to-peer membership management for gossip-based protocols. IEEE Transactions on Computers 52: 139– 149.

[26] Pittel B (1987) On Spreading a Rumor. SIAM Journal on Applied Mathematics 47: 213–223.

[27] Eugster PT, Guerraoui R, Kermarrec AMM, Massoulie L (2004) Epidemic information dissemination in distributed systems. Computer 37: 60–67.

[28] Dimakis AG, Sarwate AD, Wainwright MJ (2006) Geographic gossip: efficient aggregation for sensor networks. In: 2006 5th International Conference on Information Processing in Sensor Networks. IEEE, pp. 69–76.

[29] Dimakis AD, Sarwate AD, Wainwright MJ (2008) Geographic Gossip: Efficient Averaging for Sensor Networks. IEEE Transactions on Signal Processing 56: 1205–1216.

[30] Aysal TC, Yildiz ME, Sarwate AD, Scaglione A (2009) Broadcast Gossip Algorithms for Consensus. IEEE Transactions on Signal Processing 57: 2748–2761.

[31] Kashyap A, Basar T, Srikant R (2006) Consensus with Quantized Information Updates. In: Proceedings of the 45th IEEE Conference on Decision and Control. IEEE, pp. 2728–2733.

[32] Yildiz M, Pagliari R, Ozdaglar A, Scaglione A (2010) Voting models in random networks. In: Information Theory and Applications Workshop (ITA), 2010. pp.1–7. doi:10.1109/ITA.2010.5454090.

[33] Chandra TD, Griesemer R, Redstone J (2007) Paxos made live: An engineering perspective. In: Proceedings of the Twenty-sixth Annual ACM Symposium on Principles of Distributed Computing. New York, NY, USA: ACM, PODC ′07, pp. 398–407. doi:10.1145/1281100.1281103. Available: http://doi.acm.org.ezproxy.net.ucf.edu/10.1145/1281100.1281103.

[34] Mocanu A, Bădică C (2014) Bringing paxos consensus in multi-agent systems. In Proceedings of the 4th International Conference on Web Intelligence, Mining and Semantics (WIMS14). New York, NY, USA: ACM, WIMS ′14, pp. 51:1–51:6. doi:10.1145/2611040.2611099. Available: http://doi.acm.org.ezproxy.net.ucf.edu/10.1145/2611040.2611099.

[35] Raghavan UN, Albert R, Kumara S (2007) Near linear time algorithm to detect community structures in large-scale networks. Phys Rev E 76: 036106. 10.1103/PhysRevE.76.036106 17930305

[36] Castellano C, Fortunato S, Loreto V (2009) Statistical physics of social dynamics. Reviews of Modern Physics 81: 591.

[37] Sood V, Redner S (2005) Voter model on heterogeneous graphs. Physical Review Letters 94: 178701. 15904343 10.1103/PhysRevLett.94.178701

[38] Schweitzer F, Behera L (2009) Nonlinear voter models: the transition from invasion to coexistence. The European Physical Journal B 67: 301–318.

[39] Hagberg AA, Schult DA, Swart PJ (2008) Exploring network structure, dynamics, and function using NetworkX. In: Proceedings of the 7th Python in Science Conference (SciPy2008). Pasadena, CA USA, pp. 11–15.

[40] Oliphant TE (2007) Python for scientific computing. Computing in Science & Engineering 9.

[41] Newman MEJ, Watts DJ (1999) Renormalization group analysis of the small-world network model. Physics Letters A 263: 341–346.

[42] Hollander CD, Wu AS (2011) The current state of normative agent-based systems. Journal of Artificial Societies and Social Simulation 14.

